# Resistance Pattern of Antibiotics in Patient Underwent Open Heart Surgery with Nosocomial Infection in North of Iran

**DOI:** 10.5539/gjhs.v8n2p288

**Published:** 2015-08-05

**Authors:** Alireza Davoudi, Narges Najafi, Shahriar Alian, Atefe Tayebi, Fatemeh Ahangarkani, Samaneh Rouhi, Amirhosein Heydari

**Affiliations:** 1 Antimicrobial Resistance Research Center, Department of Infectious Diseases, Mazandaran University of Medical Sciences, Sari, Iran; 2 Student Research Committee, Kurdistan University of Medical Sciences, Sanandaj, Iran; 3 Cellular & Molecular Research Center and Department of Microbiology, Kurdistan University of Medical Sciences, Sanandaj, Iran

**Keywords:** nosocomial infection, antibiotic resistance, open heart surgery, ESBL, MRSA

## Abstract

**Background:**

Patients who undergo cardiac surgery appear to be at increased risk for the development of Nosocomial infections (NIs). The development of antibiotic-resistant infections has been associated with significantly greater hospital mortality rates compared to similar infections caused by antibiotic-sensitive pathogens.

**Objectives:**

The purpose of this study is survey of Nis and antibiotic resistance patterns of causative bacteria among patients who underwent open heart surgery in the north of Iran during a 2-year period between September 2012 and September 2014.

**Methods:**

In this cross-sectional study we evaluated 187 patients that underwent open heart surgery with NIs. Demographic feature, clinical characteristics and risk factors of each infection were recorded. The antibiotic susceptibility test was performed using the Minimum inhibitory concentration (MIC) method according to the standard protocol of Clinical & Laboratory Standards Institute (CLSI). Detection of Extended-spectrum beta-lactamase (ESBL) producing bacteria was performed by the double-disk synergy (DDS) test; also Methicillin-resistant Staphylococcus (MRSA) strains were identified by MRSA Screen Agar. The collected data were analyzed using the SPSS software (ver. 16) and, descriptive statistics were used.

**Results:**

Out Of total of 2253 hospitalized patients who underwent open heart surgery, 187(5.05%) patients had NIs. 51.9% of the patients were female. The rates of surgical site infection (SSI), respiratory tract infection, endocarditis, Urinary tract infection (UTI), blood Infection and mediastinitis were 27.80, 25.66%, 17.64, 17.11% 8.55% and 3.20% respectively. E.coli and S.aureus were the most causative agents of NIs. The rate of ESBL-producing bacteria was 14.28- 71.42% among enterobacteriaceae and the rate of MRSA was 54.2% among S.aureus strains. All isolated Acinetobacter.spp were Multi-drug resistance (MDR).

**Conclusions:**

We showed that the rate of NIs among these high-risk patients was in the average level. But the emergence of MRSA and ESBL bacteria is increasing in our region.

## 1. Introduction

Nosocomial infections (NIs) pose a serious threat to patients, especially in the high-risk wards (Ding et al., 2009). Globally, 8.7 % of the hospitalized patients are affected with NIs (Behzadnia et al., 2014). NIs after open heart surgery represent serious complications corresponding with significant morbidity, mortality, increased need for antimicrobial therapy and economic burden (Lola et al., 2011). NIs have been reported to occur in 5–21% of cardiac surgery patients and in these patients, infections are serious and often life-threatening complications (Kollef et al., 1997; Michalopoulos et al., 2006). Patients who undergo cardiac surgery appear to be at increased risk for the development of NIs due to the presence of multiple surgical wounds, frequent postoperative utilization of invasive devices, and the common use of antibiotics in the perioperative period. Additionally, current administration practices of antibiotics may help to explain the emergence of NIs due to antibiotic resistant pathogens in this and other groups of critically ill patients. The development of such antibiotic-resistant infections has been associated with significantly greater hospital mortality rates compared to similar infections caused by antibiotic sensitive pathogens. Common problem in the treatment of NIs is the increase of antibiotic-resistant organisms (Kollef et al., 1995; Goldmann et al., 1996; Rahmati et al., 2014). Prevention of NIs remains of the uttermost importance. Identifying NIs risk factors and antibiotic resistance pattern of causative agents which vary between populations, is important in preventing infection in any patient who underwent open heart surgery. To the best of our knowledge this study is the first surveillance on patients that underwent open heart surgery with NIs in the north of Iran. The purpose of this study is survey of NIs and antibiotic resistance patterns of causative bacteria among patients who underwent open heart surgery in the Fatemeh Zahra teaching hospital to help the physicians choose better antibiotics in the start of empiric therapy.

## 2. Material and Methods

### 2.1 Study Population and Design

This is a cross-sectional study. This study was approved by the Ethics Committee of Mazandaran University of Medical Sciences (Code No: 9135, Date: July 11, 2012). All the patients with NIs who were admitted to Fatemeh Zahra teaching hospital (a governmental heart surgery center in Mazandaran province, northern Iran) in the period between September 2012 and September 2014 were studied. All these patients underwent open heart surgery. The census method was used for sampling. NIs were defined based on the National Directory of Nosocomial Infections Surveillance System (Masoumi, 2007). We gathered information that included demographic and clinical characteristics, risk factors, and medical history, and main diagnosis, types of NIs and clinical samples of the patients that had NIs.

### 2.2 Clinical Sample Collection and Identification of Bacteria

Samples included deep tissue infected through the wounds of surgical sites, sputum, tracheal secretions, wound debridement of infected tissue, drainage of mediastinum infected tissues, urine and blood. Sampling was done by the head nurse and the samples were immediately transported in a transport medium to the hospital’s microbiology laboratory (To prevent the loss of some of the microorganisms present in the samples that take some time to culture and stain in the laboratory; we used Stuart transport medium). Identification of the organisms causing infection was performed according to the standard microbiological procedures (Koneman et al., 1997; Collee et al., 1996).

### 2.3 Antimicrobial Susceptibility Test

Susceptibility of the clinical isolates to routinely used antibiotics was determined by the standard broth dilution (micro dilution broth) technique. MIC is defined as the lowest concentration of an antibiotic that inhibits the visible growth of bacteria after overnight culture. The bacterial suspension equivalent to 0.5 McFarland standards was prepared provided in Müller-Hinton Broth (MHB) (Merck, Germany). The final standard concentration of the bacteria was increased to 5×10^5^ CFU/ml. Serial concentrations of antibiotics were prepared from 512 µgr/ml to 1 µgr/ml. MIC was determined according to the recommendations of the standard protocol of CLSI(CLSI, 2010). The antibiotics that were used were Ceftriaxone, Ciprofloxacin, Gentamicin, Clindamycin, Oxacillin, Penicillin, Cotrimoxazole, Ampicillin, Vancomycin, Amikacin, Ceftazidime, Imipenem, Norfloxacin, Cefazolin and Novobiocin. The antibiotics were purchased from Sigma chemical company.

#### 2.3.1 Detection of Extended-Spectrum Beta-Lactamase (ESBL) Producing Enterobacteriaceae

ESBL-producing enterobacteriaceae was detected using the double-disk synergy (DDS) test (Ramazanzadeh, 2010, Jarlier et al., 1988). ESBL presence was assayed using the following antibiotic disks (MAST, UK): cefotaxime (30 μg), cefotaxime/clavulanic acid (30/10 μg), ceftazidime (30 μg), and ceftazidime/clavulanic acid (30/10 μg). Escherichia coli ATCC 25922 strains served as positive control.

#### 2.3.2 Detection of Methicillin-Resistant Staphylococcus (MRSA) Strains

For detection of methicillin-resistant Staphylococcus strains, Oxacillin Screen Agar (originally named MRSA Screen Agar) was used. Staphylococcus strains were cultured on Muller Hinton agar containing 4% NaCl and 6 mg/L oxacillin and were incubated for 24 hours.

### 2.4 Statistical Analysis

The collected data were analyzed using SPSS software (ver. 16). Descriptive statistics were used.

## 3. Results

Out of total of 2253 hospitalized patients with open heart surgery in the north of Iran during the 2-year period between September 2012 and September 2014, 187(5.05%) had NIs from which 90(48%) were female and 97(51.9%) were male. The average age was 63.45±14.14 years old. The average duration of hospitalization was 7.32±3.42 days.

The prevalence of surgical site infection (SSI), respiratory tract infection, endocarditis, urinary tract infection (UTI), blood Infection and mediastinitis were 27.80%, 25.66%, 17.64%, 17.11%, 8.55% and 3.20% respectively.

The demographic feature, risk factors and clinical characteristics of every infection have been demonstrated in table 1 and 2; the incidence causative agent of NIs are portrayed in table 3 and antibiotic resistance pattern of the bacteria that cause NIs are given in tables 4 and 5.

**Table 1:** Demographic feature and risk factors of infection

		Respiratory tract Infection No. (%)	SSI No. (%)	UTI No. (%)	Blood Infection No. (%)	Mediastinitis No. (%)	Endocarditis No. (%)	Total No. (%)
Gender	Female	22(45.5)	22(42.3)	22(68.78)	8(50)	1(16.7)	16(48.5)	90(48.)
	Male	26(54.5)	30(57.7)	10(31.25)	8(50)	5(83.3)	17(51.5)	97(51.9)
Age	Year	63.75±13.81	65±9.2	68±12.68	67.7±16.25	57±4.18	55.96±18.12	63.45±14.14
Average duration of hospitalization	Day	7.1±2.2	6.2±1.7	7.1±5.23	6.93±1.65	8.5±3.67	8.15±6.2	7.32±3.42
Risk factor	Diabetes	15(34.1)	26(50)	8(25)	9(56.3)	6(100)	11(33.3)	83(44.4)
	HTN	24(54.5)	29(55.8)	19(59.37)	10(62.5)	4(66.7)	8(24.2)	94(50.3)
	Cardiovascular disease	21(31.7)	22(42.3)	11(34.37)	8(50)	4(66.7)	5(15.2)	71(38)
	Urine catheter	13(27.8)	13(25)	23(71.87)	7(43.8)	6(100)	9(27.3)	101(54)
	Central venous device	0	0	0	0	3(50)	2(6.06)	5(2.67)
	Mechanical intubation	41(93.2)	12(23.1)	13(40.62)	5(31.3)	6(100)	8(24.2)	85(45.5)
	Steroid therapy	1(2.3)	0	3(9.37)	0	0	4(12.1)	8(4.3)
	Lung diseases	5(11.4)	11(21.2)	6(18.75)	0	0	2(6.1)	24(12.7)
	Liver Cirrhosis	2(4.5)	0	2(6.25)	0	0	0	4(2.1)
	Renal failure	6(13.6)	1(1.9)	5(15.62)	3(18.8)	0	2(6.1)	17(9.1)
	Malignancy	0	0	0	0	0	0	0
	Chemotherapy	0	0	0	0	0	0	0
	Transplantation	0	0	3(1.6)	0	0	0	3(1.6)

**Table 2:** Clinical characteristics of infection

		Respiratory tract Infection No. (%)	SSI No. (%)	UTI No. (%)	Blood Infection No. (%)	Mediastinitis	Endocarditis	Total No. (%)
Symptoms	Fever	35(79.5)	13(25)	24(75)	15(93.8)	4(66.7)	5(15.2)	96(51.3)
	Dysuria	0	0	16(8.6)	0	0	0	16(8.6)
	Frequent urination	0	0	16(8.6)	0	0	0	16(8.6)
	Flank pain	0	0	4(2.1)	0	0	0	4(2.1)
	Suprapobic pain	0	0	3(1.6)	0	0	0	3(1.6)
	Nausea	0	0	0	0	0	1(.3)	1(.3)
	Vomiting	0	0	0	0	0	1(.3)	1(.3)
	Chest pain	5(11.4)	1(1.9)	0	1(6.3)	0	1(.3)	8(4.3)
	Cough	9(20.5)	0	0	0	0	1(.3)	10(5.3)
	Increase of sputum	39(88.6)	0	3(1.6)	0	6(100)	0	48(25.7)
	Dyspnea	32(72.7)	1(1.9)	6(3.2)	4(25)	4(66.7)	1(.3)	48(25.7)
	Wound erythema	0	40(76.92)	0	0	4(66.7)	0	44(23.4)
	Wound oozing	0	39(75)	1(3.12)	0	4(66.7)	0	44(23.4)
	Suture openings	0	16(30)	0	0	0	0	16(8.6)

**Table 3:** Causative agent of Nis

Causative agent by site				No.(%) episodes of infection			

NO	SSI NO (%) 52(27.8)	Respiratory Infection NO (%) 48(25.6)	UTI NO (%) 32(17.1)	Blood Infection NO (%) 16(8.55)	Mediastinitis NO (%) 6(3.2)	Endocarditis NO (%) 33(17.6)	Total (N=187) NO (%)
*P. aeruginosa*	2(3.84)	8(16.66)	2(6.25)	0	0	0	12(13.79%)
*Acinetobacter.spp*	1(1.92)	3(6.25)	1(3.12)	0	1(16.6)	1(3.03)	7(8.04%)
*E.coli*	7(13.46)	6(12.5)	16(50)	0	0	0	29(33.33%)
*Enterobacter*.spp	3(5.76)	2(4.16)	3(9.37)	0	0	0	8(9.19%)
*Klebsiella.spp*	3(5.76)	0	3(9.37)	1(6.25)	0	0	7(8.04%)
*S. aureus*	14(26.92)	5(10.41)	0	6(37.5)	3(18.75)	5(9.09)	33(37.93%)
*S. epidermidis*	1(1.92)	0	1(3.12)	0	0	0	2(2.29%)
Enterococci	0	0	0	0	0	1(3.03)	1(1.14%)
Culture negative	21(4.3)	20(41.66)	6(18.75)	9(56.25)	2(33.33)	26(78.78)	88(47.1%)

**Table 4:** Antibiotic resistance pattern of gram negative bacteria isolated from infection

	*P.aeuroginosa* NO.(%)			*Acinetobacter.spp* NO.(%)			*E.coli* NO.(%)			*Enterobacter .spp* NO.(%)			*Klebsiella.spp* NO.(%)		

Antibiotics	S	I	R	S	I	R	S	I	R	S	I	R	S	I	R
Ceftriaxone	0	10 (83.3)	2 (16.6)	0	0	7 (100)	3 (10.3)	24 (82.75)	2 (6.89)	4 (50)	3 (37.5)	1 (12.5)	0	3 (37.5)	4 (57.1)
Ceftazidime	2 (16.6)	4 (33.3)	6 (50)	0	0	7 (100)	3 (10.3)	18 (62.06)	8 (27.5)	0	3 (37.5)	5 (62.5)	1 (14.2)	1 (14.2)	5 (71.42)
Ciprofloxacin	2 (16.6)	8 (66.6)	2 (16.6)	0	0	7 (100)	3 (10.3)	24 (82.7)	2 (6.89)	3 (37.5)	3 (37.5)	2 (25)	1 (14.2)	2 (28.5)	4 (57.1)
Gentamicin	10 (83.3)	0	2 (16.6)	0	0	7 (100)	25 (86.2)	2 (6.89)	2 (6.89)	3 (37.5)	2 (25)	3 (37.5)	3 (37.5)	1 (14.2)	3 (42.85)
Amikacin	10 (83.3)	2 (16.6)	0	0	0	7 (100)	27 (93.1)	0	2 (6.89)	3 (37.5)	2 (25)	3 (37.5)	4 (57.1)	0	3 (42.85)
Imipenem	10 (83.3)	0	2 (16.6)	0	0	7 (100)	23 (79.3)	2 (6.89)	4 (13.8)	2 (25)	1 (12.5)	5 (62.5)	3 (37.5)	1 (14.2)	3 (42.85)
Novobiocin	0	10 (83.3)	2 (16.6)	0	0	7 (100)	8 (27.58)	19 (65.51)	2 (6.89)	1 (12.5)	2 (25)	5 (62.5)	3 (42.85)	2 (28.5)	2 (28.5)

**Table 5:** Antibiotic resistance of gram positive bacteria isolated from infection

	*S.aureus* NO (%)			*S.epidermidis* NO (%)		

	S	I	R	S	I	R
Ampicillin	4(12.9)	15(48.38)	12(38.70)	0	2(100)	0
Penicillin	0	19(61.29)	12(38.70)	0	2(100)	0
Oxacillin	2(6.45)	3(9.67)	26(83.87)	0	0	2(100)
Vancomycin	30(96.77)	0	1(3.22)	2(100)	0	0
Clindamycin	0	19(61.29)	12(38.70)	2(100)	0	0
Co-trimoxazole	0	19(61.29)	12(38.70)	0	0	2(100)
Norfloxacin	7(22.58)	12(38.70)	19(61.29)	2(100)	0	0
Cefalotin	17	7(22.58)	7(22.58)	0	2(100)	0

The prevalence of ESBL bacteria for P. *aeruginosa*, *Acinetobacter*.spp, *E.coli*, *Enterobacter*.spp and *Klebsiella* .spp were 4(33.3%), 5(71.42%), 8(27.58%), 2(25%) and 1(14.28%) respectively. The rates of MRSA Staphylococcus strains for *S.aureus* and *S. epidermidis* were 26(54.2%) and 2(100%)

## 4. Discussions

Despite advances in antisepsis, asepsis, antibiotic prophylaxis and in surgical techniques, NIs continue to complicate after surgery in many patients (Segers et al., 2006). The rate of NIs in our study was 8.3%. Lomtadze et al. and Levy et al. reported the incidence of NIs in about 16% of patients that underwent heart surgery (Segers et al., 2006; Lee et al., 2010). However the rate of NIs in Sarvikivi et al study was 25 % (Sarvikivi et al., 2007). The rate of NIs after heart surgery in the study of Kollef, Sharpless et al. conducted in 1997 was 5-21% (Kollef et al., 1997). Michalopoulos and colleagues reported that 5% of patients developed microbiologically documented NIs after open cardiac surgery which is close to our findings (Michalopoulos et al., 2006).

SSI accounts for 15% of all NIs among surgical patients (Lee et al., 2010). SSI was diagnosed in 3% of the patients in Lepelletier et al study but in 13.5% in Lee et al study that was performed in Taiwan (Lee et al., 2010; Lepelletier et al., 2005). The most causative agent of SSI was *S.aureus* in our study. Similarly, this agent was the most common organism isolated in Mundhada et al. research (Mundhada & Tenpe, 2015). Lemaignen et al. reported 4.1% occurrence of SSIs after cardiac surgery and, *S.aureus* was the most prevalent microorganism associated with them (Lemaignen et al., 2015). Risk factors for SSI were older age and diabetes in our study. Diabetes is one of the risk factors for the development of postoperative Nis in surgical patients. Patients with diabetes are more susceptible to many types of infection, including NIs and this has been the findings of Yamashita et al and Vardakas et al. (Yamashita et al., 2000; Vardakas et al., 2007). We observed that 4.3% of SSI was negative culture. The appearance of postoperative SSI in the absence of cultivable bacterial pathogens is a usual dilemma for the surgeons. Culture-negative SSIs happen because of prior antimicrobial therapies, the presence of fastidious or slow-growing bacteria such as Legionella spp, mycobacteria and Mycoplasma spp and infections caused by mundane bacteria that may be dismissed as contaminants, etc. (Rasnake & Dooley, 2006; Reddy, 2012).

Riera et al in Spain showed the rate of nosocomial pneumonia was 1.2% and the main risk factors for respiratory tract infections were left ventricular ejection fraction (Riera et al., 2010). Lomtadze et al in Georgia reported that the rate of nosocomial respiratory tract infections was 7% after heart surgery. Consistent with our results, Mechanical ventilation was one of the most common risk factors in Lomtadze et al study (Lomtadze et al., 2010; Lemaignen et al., 2015). *P. aeruginosa* and *S. aureus* were the most found bacteria isolated in pneumonia. In NIs pneumonia, *P. aeruginosa*, *A. baumannii*, and the Enterobacteriaceae are the main bacteria (Anton et al., 2010). We showed that 41.66% of pneumonia patients had negative culture results. Negative upper airway swab, alternative sampling methods such as induced sputum or broncho alveolar lavage may cause negative culture in NIs pneumonia (Ahmed et al., 2014).

Nosocomial endocarditis is associated with total parenteral nutrition lines, presence of intravenous catheters, pacemakers, etc. (Kasper & Harrison, 2005). In our study, 24.4% of the patients with nosocomial endocarditis had mechanical intubation and most causative bacteria were S. aureus. *Enterococci* and *Streptococcus bovis* were the most frequent bacteria in Barrau et al study (Barrau et al., 2004). Close to our findings, Takayama found *S. aureus* was responsible for endocarditis in 21.3% of the cases (Takayama et al., 2010). Close to 80% of endocarditis were negative culture, because some germs do not grow well in a laboratory setting and some people have taken antibiotics in the past that keep germs from growing.

The rate of UTI was 17.1% in our study and the main risk factor was urine catheter that was used on our patients. In a study that was conducted by Sodano et al in Italy, the rates of catheter-associated and device-associated UTIs in heart surgery patients were 5.8% and.6% respectively (Sodano et al., 2004). Also Mirinazhad et al. in Iran showed that all the patients had NIs associated with positive urine culture related to catheter-associated UTI. The most common microorganisms that Mirinazhad et al isolated were *Enterobacter spp*. (37.5%) (Mirinazhad et al., 2011). However *E.coli* was the most common bacteria isolated in our study. According to many studies in Iran, the main cause of UTI is still *E. coli* (Aminizadeh & Kashi, 2011; Behzadnia et al., 2014; Rezai et al., 2015; Saffar et al., 2008; Davoudi et al., 2012). In our study, 18.7% of the patients with UTI had negative culture.

Lomtadze et al. found the rate of blood infection to be 7.75% which is consistent with our results (Lomtadze et al., 2010). Barker reported 0.3% mediastinitis and 0.09% endocarditis in pediatric patients after cardiac surgery (Barker et al., 2010). Al-Hazmi showed *E. coli*, Enterococcus and *Pseudomonas* are the most common organisms isolated from blood infections (Al-Hazmi et al., 2014). In our study, the most prevalent bacteria isolated from mediastinitis was *S.aureus* as it was in the study of Ghotaslou et al which is similar to our findings (Ghotaslou et al., 2008).

In our study, among Gram-negative enterobacteriaceae, *E.coli* and *P. Aeruginosa*, and among Gram-positive bacteria *S. aureus* was the most common isolated bacteria. Lavakhamseh et al. showed that the most common bacterium isolated from patients with NIs was *E. coli* with 68.51% rate, and the highest resistance was found for Co-trimoxazole with 57.47% incidence (Lavakhamseh et al., 2014). In our study *E.coli* and *Enterobacter*.spp have shown high levels of resistance to Ceftazidime, Imipenem, Gentamicin and Amikacin respectively. Resistance to antibiotics among *P. aeuroginosa* was 16.6% to Ceftriaxone, Ceftazidime, Ciprofloxacin, Gentamicin, Amikacin, and Imipenem. Incidence of resistance to Ceftazidime, ceftriaxone and Ciprofloxacin among *Klebsiella*.spp was 71.42%, 57.1% and 57.1% respectively. Results in our study showed all *Acinetobacter*.spp were MDR. The rate of ESBL-producing bacteria was 14.28- 71.42% in our study. ESBL-producing bacteria confer significant resistance to penicillin, aztreonam antibiotics and narrow and extended-spectrum cephalosporin. They also frequently show resistance to aminoglycosides, trimethoprim/sulfamethoxazole and quinolones (Paterson et al., 2003). In consistent with our result, Rezai et al. reported 30.5% ESBL-producing *E.coli* (Rezai et al., 2015). Also the rates of extended-spectrum beta-lactamase production in *K. pneumoniae* and *E. coli* strains were determined as 14% and 6% respectively in Surucuoglu et al. study (Surucuoglu et al., 2005). ESBL-producing isolates have been responsible for provoking outbreaks in many hospitals. Considering that these genes are located in plasmid they can spread in hospital environment.

*S. aurous* and *S.epidermidis* had high rates of antibiotic resistance. However resistance to vancomycin was observed in one isolated *S. aurous*. In our study, the rate of MRSA *S. aurous* was 54.2%. Azimian et al reported the incidence of MRSA S. aurous to be 47.5% (Azimian et al., 2012). Although Abdulgader showed that the proportion of Panton-Valentine leukocidin positive MRSA carriage infections ranged from 0.3 to 100% in humans (Abdulgader et al., 2015). MRSA is responsible for an increasing number of serious hospital and community-acquired infections. NIs with MRSA strains requires treatment with glycopeptide antibiotics which are nephrotoxic (Nelson et al., 2015; Pacheco et al., 2011).

Studies examining NIs bacteremia have shown that infection due to antibiotic-resistant pathogens is usually associated with greater patient mortality compared to the same infections attributed to antibiotic-sensitive bacteria (Chow et al., 1991; Rezai et al., 2012). It is necessary that infection control procedures be implemented carefully and antibiotic resistance patterns of organisms causing NIs should be checked periodically, specifically in heart surgery centers to guide empirical antibiotic therapies

## 5. Conclusions

Our study shows that although the rate of NIs following open heart surgery is within a reasonable limit, but MDR gram-negative bacteria and gram-positive bacteria are increasing. This issue can seriously affect the outcome of patients who underwent heart surgery. Therefore, using effective measures such as infection control, isolation of patients with MDR infections and implementation of antibiotic stewardship is recommended.

## References

[ref1] Abdulgader S. M, Shittu A. O, Nicol M. P, Kaba M (2015). Molecular epidemiology of Methicillin-resistant Staphylococcus aureus in Africa: A systematic review. Front Microbiol.

[ref2] Ahmed B, Bush A, Davies J. C (2014). How to use: Bacterial cultures in diagnosing lower respiratory tract infections in cystic fibrosis. Arch Dis Child Educ Pract Ed.

[ref3] Al-Hazmi H. H, Al-Zahrani T, Elmalky A. M (2014). Hospital acquired blood stream infection as an adverse outcome for patients admitted to hospital with other principle diagnosis. Saudi J Anaesth.

[ref4] Aminizadeh Z, Kashi M (2011). Prevalence of multi-drug resistance and pandrug resistance among multiple gram-negative species: Experience in one teaching hospital, Tehran, Iran. Int Res J Microbiol.

[ref5] Anton Y, Peleg M, Hooper D (2010). Hospital-Acquired Infections Due to Gram-Negative Bacteria. N Engl J Med.

[ref6] Azimian A, Najar-Pirayeh S, Mirab-Samiee S, Naderi M (2012). Occurrence of methicillin resistant Staphylococcus aureus (MRSA) among clinical samples in tehran-iran and its correlation with polymorphism of specific accessory gene regulator (AGR) groups. Braz J Microbiol.

[ref7] Barker G. M, O’brien S. M, Welke K. F, Jacobs M. L, Jacobs J. P, Benjamin D. K, Li J. S (2010). Major infection after pediatric cardiac surgery: A risk estimation model. Ann Thorac Surg.

[ref8] Barrau K, Boulamery A, Imbert G, Casalta J. P, Habib G, Messana T, Raoult D (2004). Causative organisms of infective endocarditis according to host status. Clin Microbiol Infect.

[ref9] Behzadnia S, Davoudi A, Rezai M. S, Ahangarkani F (2014). Nosocomial infections in pediatric population and antibiotic resistance of the causative organisms in north of Iran. Iran Red Crescent Med J.

[ref10] Chow J. W, Fine M. J, Shlaes D. M, Quinn J. P, Hooper D. C, Johnson M. P, Yu V. L (1991). Enterobacter bacteremia: Clinical features and emergence of antibiotic resistance during therapy. Ann Intern Med..

[ref11] Collee J, Miles R, Watt B (1996). Tests for the identification of bacteria. Mackie and McCartney. Practical Medical Microbiolog.

[ref12] Davoudi A. R, Najafi N, Hoseini Shirazi M, Ahangarkani F (2012). Frequency of bacterial agents isolated from patients with nosocomial infection in teaching hospitals of Mazandaran University of Medical Sciences in 2012. Caspian J Intern Med.

[ref13] Ding J. G, Sun Q. F, Li K. C, Zheng M. H, Miao X. H, Ni W, He W. F (2009). Retrospective analysis of nosocomial infections in the intensive care unit of a tertiary hospital in China during 2003 and 2007. BMC Infect Dis.

[ref14] Ghotaslou R, Yagoubi A. R, Khalili A. A, Mahmodian R (2008). Mediastinitis after cardiac surgery in Madani Heart Center, Tabriz, Iran. Jpn J Infect Dis.

[ref15] Goldmann D. A, Weinstein R. A, Wenzel R. P, Tablan O. C, Duma R. J, Gaynes R. P, Martone W. J (1996). Strategies to Prevent and Control the Emergence and Spread of Antimicrobial-Resistant Microorganisms in Hospitals. A challenge to hospital leadership. JAMA.

[ref16] Jarlier V, Nicolas M, Fournier G, Philippon A (1988). Extended broad-spectrum beta-lactamases conferring transferable resistance to newer beta-lactam agents in Enterobacteriaceae: Hospital prevalence and susceptibility patterns. Rev Infect Dis.

[ref17] Kasper D. L, Harrison T. R (2005). Harrison’s principles of internal medicine.

[ref18] Kollef M. H, Sharpless L, Vlasnik J, Pasque C, Murphy D, Fraser V. J (1997). The impact of nosocomial infections on patient outcomes following cardiac surgery. Chest.

[ref19] Kollef M. H, Wragge T, Pasque C (1995). Determinants of mortality and multiorgan dysfunction in cardiac surgery patients requiring prolonged mechanical ventilation. Chest.

[ref20] Koneman E, Allen S, Janda W, Schreckenberger R, Winn W (1997). Introduction to microbiology. Part II: Guidelines for the collection transport, processing analysis and reporting of culture from specific specimen sources.

[ref21] Lavakhamseh H, Shakib P, Rouhi S, Mohammadi B, Ramazanzadeh R (2014). A survey on the prevalence and antibiotic sensitivity of nosocomial infections in the besat hospital, SANANDAJ, IRAN. Journal NI.

[ref22] Lee Y. P, Feng M. C, Wu L. C, Chen S. H, Chen Y. H, Chiu C. C, Lu P. L (2010). Outcome and risk factors associated with surgical site infections after cardiac surgery in a Taiwan medical center. J Microbiol Immunol Infect.

[ref23] Lemaignen A, Birgand G, Ghodhbane W, Alkhoder S, Lolom I, Belorgey S, Lucet J. C (2015). Sternal wound infection after cardiac surgery: Incidence and risk factors according to clinical presentation. Clin Microbiol Infect.

[ref24] Lepelletier D, Perron S, Bizouarn P, Caillon J, Drugeon H, Michaud J. L, Duveau D (2005). Surgical-site infection after cardiac surgery: Incidence, microbiology, and risk factors. Infect Control Hosp Epidemiol.

[ref25] Lola I, Levidiotou S, Petrou A, Arnaoutoglou H, Apostolakis E, Papadopoulos G. S (2011). Are there independent predisposing factors for postoperative infections following open heart surgery?. J Cardiothorac Surg.

[ref26] Lomtadze M, Chkhaidze M, Mgeladze E, Metreveli I, Tsintsadze A (2010). Incidence and risk factors of nosocomial infections after cardiac surgery in Georgian population with congenital heart diseases. Georgian Med News.

[ref27] Masoumi A (2007). Directory of nosocomial infections surveillance system.

[ref28] Michalopoulos A, Geroulanos S, Rosmarakis E. S, Falagas M. E (2006). Frequency, characteristics, and predictors of microbiologically documented nosocomial infections after cardiac surgery. Eur J Cardiothorac Surg.

[ref29] Mirinazhad M, Chavoshinazhad M, Ghorbanian N (2011). Epidemiologic and Etiologic Evaluation of Acquired Urinary Tract Infections in Cardiac Surgery ICU Patients. J Cardiovasc Thorac Res.

[ref30] Mundhada A. S, Tenpe S (2015). A study of organisms causing surgical site infections and their antimicrobial susceptibility in a tertiary care Government Hospital. Indian J Pathol Microbiol.

[ref31] Nelson M. U, Bizzarro M. J, Baltimore R. S, Dembry L. M, Gallagher P. G (2015). Clinical and Molecular Epidemiology of Methicillin-Resistant Staphylococcus aureus in a Neonatal Intensive Care Unit in the Decade Following Implementation of an Active Detection and Isolation Program. J Clin Microbiol.

[ref32] Pacheco R. L, Lobo R. D, Oliveira M. S, Farina E. F, Santos C. R, Costa S. F, Levin A. S (2011). Methicillin-resistant Staphylococcus aureus (MRSA) carriage in a dermatology unit. Clinics (Sao Paulo).

[ref33] Paterson D. L, Hujer K. M, Hujer A. M, Yeiser B, Bonomo M. D, Rice L. B, Bonomo R. A (2003). Extended-spectrum beta-lactamases in Klebsiella pneumoniae bloodstream isolates from seven countries: Dominance and widespread prevalence of SHV- and CTX-M-type beta-lactamases. Antimicrob Agents Chemother.

[ref34] Rahmati M, Razaghi A, Doostdar H, Yaghoubi H, Masoumi S, Rezai M.-S (2014). Comparison of azithromycin, amoxicillin and amoxicillin/clavulanicacid in the treatment of children with acute bacterial sinusitis. Journal of Mazandaran University of Medical Sciences.

[ref35] Ramazanzadeh R (2010). Etiologic agents and extended-spectrum beta-lactamase production in urinary tract infections in Sanandaj, Iran. Eastern Journal of Medicine.

[ref36] Rasnake M. S, Dooley D. P (2006). Culture-negative surgical site infections. Surg Infect (Larchmt).

[ref37] Reddy B (2012). Culture negative surgical site infections. J Med Allied Sci.

[ref38] Rezai M, Ghaffari V, Abbaskhanian A, Puramiri R (2012). Comparison of once versus twice daily dose of amikacin in neonatal early sepsis. HealthMed.

[ref39] Rezai M. S, Salehifar E, Rafiei A, Langaee T, Rafati M, Shafahi K, Eslami G (2015). Characterization of Multidrug Resistant Extended-Spectrum Beta-Lactamase-Producing Escherichia coli among Uropathogens of Pediatrics in North of Iran. BioMed Research International.

[ref40] Riera M, Ibanez J, Herrero J, Ignacio Saez De Ibarra J, Enriquez F, Campillo C, Bonnin O (2010). Respiratory tract infections after cardiac surgery: impact on hospital morbidity and mortality. J Cardiovasc Surg (Torino).

[ref41] Saffar M, Enayti A, Abdolla I, Razai M, Saffar H (2008). Antibacterial susceptibility of uropathogens in 3 hospitals, Sari, Islamic Republic of Iran, 2002-2003. Eastern Mediterranean Health Journal.

[ref42] Sarvikivi E, Lyytikainen O, Nieminen H, Sairanen H, Saxen H (2007). Nosocomial infections after pediatric cardiac surgery. Am J Infect Control.

[ref43] Segers P, Speekenbrink R. G, Ubbink D. T, Van Ogtrop M. L, De Mol B. A (2006). Prevention of nosocomial infection in cardiac surgery by decontamination of the nasopharynx and oropharynx with chlorhexidine gluconate: A randomized controlled trial. JAMA.

[ref44] Sodano L, Agodi A, Barchitta M, Musumeci F, Menichetti A, Bellocchi P, Coco G (2004). Nosocomial infections in heart surgery patients: Active surveillance in two Italian hospitals. Ann Ig.

[ref45] Surucuoglu S, Gazi H, Kurutepe S, Ozkutuk N, Ozbakkaloglu B (2005). Bacteriology of surgical wound infections in a tertiary care hospital in Turkey. East Afr Med J.

[ref46] Takayama Y, Okamoto R, Sunakawa K (2010). Definite infective endocarditis: Clinical and microbiological features of 155 episodes in one Japanese university hospital. J Formos Med Assoc.

[ref47] Vardakas K. Z, Siempos Ii, Falagas M. E (2007). Diabetes mellitus as a risk factor for nosocomial pneumonia and associated mortality. Diabet Med.

[ref48] Yamashita S, Yamaguchi H, Sakaguchi M, Satsumae T, Yamamoto S, Shinya F (2000). Longer-term diabetic patients have a more frequent incidence of nosocomial infections after elective gastrectomy. Anesth Analg.

